# Identifying the natural and anthropogenic factors influencing the spatial disparity of population hollowing in traditional villages within a prefecture-level city

**DOI:** 10.1371/journal.pone.0249448

**Published:** 2021-04-02

**Authors:** Sheng Liu, Ming Bai, Min Yao, Ke Huang

**Affiliations:** 1 Zhejiang University City College, Hangzhou, Zhejiang, China; 2 College of Civil Engineering and Architecture, Zhejiang University, Hangzhou, China; 3 Zhejiang University Urban-Rural Planning & Design Institute, Hangzhou, China; Institute for Advanced Sustainability Studies, GERMANY

## Abstract

In developing countries, the phenomena of rural depopulation have been an intense continuing, which have become a major bottleneck for the sustainable revitalization of traditional villages. However, the factors influencing the spatial disparity of population hollowing (SDPH) in traditional villages within a prefecture-level city have not been fully quantitatively researched. Based on the factors that influence general villages, this study incorporated historical and cultural factors related to traditional village characteristics to construct a targeted influencing factor index system and then identified the key factors by applying the geo-detector method. With the percentage of resident population (PRP) used as a metric, this study examined Lishui, one of China’s traditional village agglomeration regions, as an example to explore SDPH in traditional villages. The results of this study were revealed in the following. (1) The average PRP value in traditional villages in Lishui was 0.68, with clear spatial disparities between the northern region (0.73) and the southern region (0.57). (2) The factors driving the SDPH included both natural and anthropogenic factors; of these, altitude, the number of public facilities, and the number of communication base stations were the most significant influencing factors. In contrast, historical and cultural factors have relatively low impacts. (3) The interaction relationships of pair factors were often enhanced on a bivariate basis, with the highest enhanced impact occurring from the interaction of two variables: the degree of intangible cultural inheritance and altitude. (4) The intervals of the variables leading to the hollowing of the population above a moderate level can be detected. This method can effectively analyze the factors influencing SDPH in traditional villages; can help reveal the interaction impact of pair factors; and can help identify the factors’ risk intervals.

## 1. Introduction

### 1.1. Background

Protecting and utilizing traditional villages have long been valued globally, and villages with high heritage protection value have been included in the World Heritage List since 1972 [1]. However, population hollowing is widespread in rural areas in developing countries [2], and most traditional villages continue to suffer from rapid population loss and aging under the joint impact of fewer support funds and urbanization. Many laborers in these villages go to cities or towns to work, with some returning home during the harvest season or long holidays [3]. This population outflow or decrease in a certain territory is called population hollowing [4] or rural depopulation [5], and can lead to agro-farming depressions, uninhabited housing, a lack of inheritance in historical relics, and the disappearance of entire villages [6,7]. This has become a major bottleneck for the sustainable revitalization of traditional villages or historical heritage [8,9].

Population hollowing in traditional villages is particularly critical in China [10]. The degree of population hollowing in villages is still with a significant spatial disparity in the same city. For instance, the degree of population hollowing in traditional villages in the outer suburbs has exceeded that of the suburb traditional villages in Yangquan city, Shanxi Province [11], and that population hollowing in mountain areas is more significant than in valley areas in Hunan Province [12].

Since 2012, China has identified 6819 national traditional villages in five batches [13]. Specific developments and rationalizations of these villages are implemented by governments of their local cities and counties, which could offer support policies, funds, and regional tourism plannings [8,14]. However, at the county level, the number of traditional villages is relatively small, and therefore, counties still merely focus on their matters and are often unable to scientifically understand the big picture of the spatial disparity of population hollowing (SDPH) of traditional villages. In contrast, a prefecture-level city, as the main administrative unit in China (a collection of districts and counties, including urban and rural areas [15]), is a comparatively suitable research scope. On the one hand, many prefecture-level cities already have a large number of national traditional villages (as of 2018, there are 20 prefecture-level cities with more than 80 national traditional villages). On the other hand, this administrative unit is the major local governmental body to establish important rural development policies, fund subsidy arrangement, regional infrastructure, and tourism planning. Thus, the research results in this scope will be strongly applicable, since the targeted prevention and control strategies can be directly implemented based on scientific analysis.

- Studies have shown that urbanization is often a driving force for depopulation around developed areas; however, the spatial disparity of population hollowing (SDPH) in traditional villages within a prefecture-level city cannot be fully explained by the urban pull [16]. It is urgent to explore the specific key driving factors to provide a scientific basis for strategies to prevent population loss. Specifically, for the specific cities, such research is needed to answer the following questions: (1) Which factors mainly lead to SDPH? (2) Which factors have a greater interaction? (3) What are the risk impact intervals of each factor?

### 1.2. Literature review

Recently, many studies have explored the factors driving SDPH in villages. Some studies have found that natural, economic, and social factors have played an important role, including temperature, altitude, and slope at the natural level [17,18]; facilities and transportation, employment, education, and construction capacity at the social level [19]; and per capita income, collective income, and fixed asset investment at the economic level [20]. Besides, some studies have focused on individual influencing factors. These studies have found that rainfall has little impact on rural population migration [21], but that telecommunications do have an important impact on population migration [22].

These explorations have provided many insights for understanding the causes of rural population migration. However, few studies have researched factors from the perspective of historical and cultural inheritance, with only a few qualitative analyses and strategic recommendations noting the importance of these factors in preventing and controlling rural population loss [11,18]. Therefore, quantitative analysis is still needed.

Besides, there are differences in the factors driving the population hollowing or migration in different kinds of villages. In economically developed American villages, villagers generally have a low willingness to move to cities, and employment has a significantly lower influence on population migration compared to social satisfaction and community attachment [23]. In Denmark, where people have higher happiness indexes and living standards, the population distribution in rural villages is affected by materials, economy, and labor. In contrast, the population distribution in suburban villages is mainly affected by reputation [24]. However, in the impoverished mountainous areas in China, rural population changes are significantly affected by economic opportunities, education demands, transportation costs, and subsistence agriculture [19]. However, only a few quantitative studies have analyzed the phenomenon of population hollowing in traditional villages with historical and cultural characteristics. Some studies have conducted driving factor researches based on a single or a small number of traditional villages [10,25], and some have made quantitative analyses on the degree of population hollowing in traditional villages in a region [11,26]. However, there have been few effective mathematical analyses of the factors affecting spatial differentiation, because it is difficult to obtain accurate data, including social, economic, and cultural statistics, for a large sample of traditional villages.

Concerning related influencing factor analysis methods, the statistical regression model is commonly used; methods mainly include multiple linear regression (MLR) [10,24] and geographically weighted regression (GWR) [12,27]. However, the traditional regression model is only effective when the variables and independent variables follow a normal distribution. In reality, these conditions are rarely met, and regression models do not quantify the influence of the interaction of independent variables. In Contrast, the geo-detector method is a robust and straightforward method to quantify the influences of driving factors and their interactions, which does not have to follow restrictedly the assumptions of traditional statistical methods [28]. It has successfully quantified the influence of the factors driving the spatial disparity of populations [29,30], and may become an effective tool to detect the SDPH of traditional villages.

### 1.3. Research aims and contents

The main objective of this study was to establish a scientific approach to investigate the key factors that influence the SDPH of traditional villages within a prefecture-level city, which can provide a basis for efficient prevention and control strategies. Therefore, from the view of Natural and Anthropogenic aspects, this study innovatively incorporated historical and cultural factors that were particular to traditional villages to construct a targeted framework of potential influencing factors. And then, taking Lishui, a prefectural-level city, as the study area, we examined its 150 traditional villages’ significant SDPH features through global autocorrelation and screened out the influencing factors, the interaction between factors, and the factors’ risk intervals by applying a correlation analysis and the geo-detector method.

## 2. Study area and data sources

### 2.1. Study area

**Fig 1:**
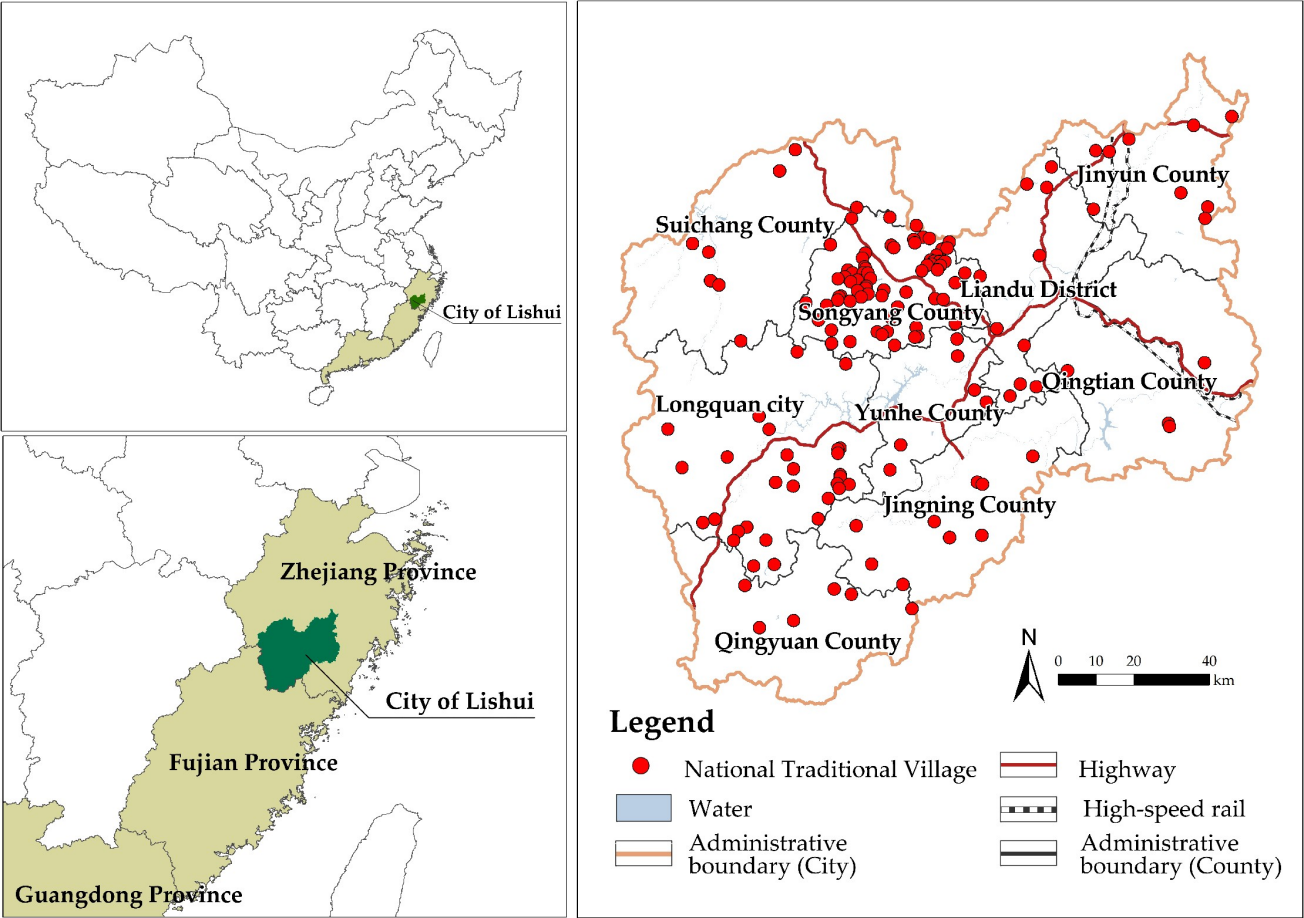
Location of Lishui and its national traditional villages. The maps were generated by ArcGIS 10.6 and were for illustrative purposes only.

In China, Zhejiang, Fujian, and Guangdong are the three provinces with the highest degree of population hollowing in villages [31]. As the research aim is to study the SDPH within a prefecture-level city, we chose Lishui city in Zhejiang Province, as the study area. It has 150 national-level traditional villages under the jurisdiction of one municipal district, one county-level city, and seven counties(Fig 1), and is the prefecture-level city area with the third-most national traditional villages in China [32]. Under the influence of external urbanization in the Yangtze River Delta, there has been a significant population hollowing of the traditional villages in Lishui, with clear differences in spatial distribution. The study area is dominated by mountainous and hilly landforms. It belongs to the mid-subtropical monsoon climate zone, with an annual average temperature of 17.9°C. The lowest average temperature is 6.7°C in January and the highest average temperature is 28.4°C in July. Most traditional villages there generally rely on traditional farming. A few villages have developed tourism to boost the local economy [33].

### 2.2. Data sources

(1) Slope and elevation data were collected using digital elevation model (DEM) data with a precision of 30 m, from the Geographical Information Monitoring Cloud Platform (www.dsac.cn) in China. (2) The Normalized Difference Vegetation Index (NDVI) data and summer and winter surface temperature data were obtained from landsat8 remote sensing data with a precision of 30 m from the US Geological Survey website (http://earthexplorer.usgs.gov). We selected the remote sensing images from July 15, 2017, to inverse the NDVI index and surface temperature in summer, and the images from January 13, 2017, to inverse the surface temperature in winter. (3) Facility data were collected from the Point of Interest (POI) data of the Gaode map in 2017 (http://lbs.amap.com/); the name, category, longitude, and latitude information were extracted, and the following location data were screened out: village, agricultural bases, scenic spots, high-speed rail stations, and relevant public service facilities. (4) The statistical data of resident population, household registered population, construction land area, collective income, per capita income, the cultural inheritance of tangible and intangible were collected from the Chinese Traditional Village Survey Registration Form (2017) of the Lishui Housing and Urban-Rural Development Bureau. (5) Communication base station data came from the base station database of the three major telecommunication operators (China Mobile, China Telecom, and China Unicom) (2017).

## 3. Method

### 3.1. Study approach

**Fig 2:**
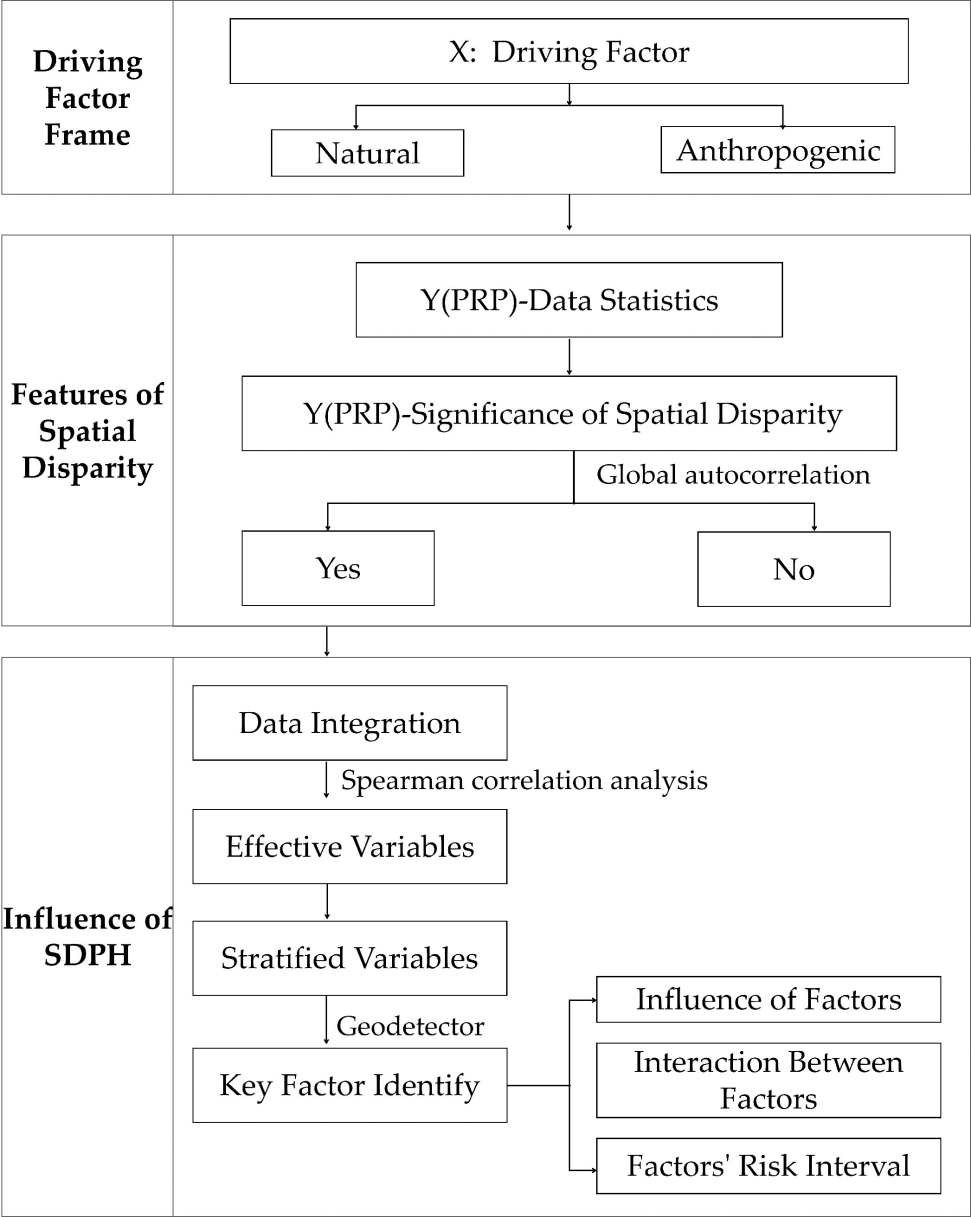
Technical route.

The characteristics and influencing factors of SDPH in traditional villages were explored in stages (Fig 2). First, a potential driving factor framework from both anthropogenic and natural aspects was established. Second, global autocorrelation was used to explore whether the percentage of resident population (PRP) in the area was related to space. Next, effective variables were screened out using the Spearman’s correlation analysis [34]. Finally, according to the requirements of the geo-detector, we stratified the effective variables and applied them into the geo-detector to identify the key factors, followed by the analysis of factors’ influences, interactions, and risk intervals.

### 3.2. Measurement of the degree of population hollowing

To measure the degree of the population hollowing, many studies have used statistical indicators of the percentage of children and the elderly, and the percentage of employees [10,35,36]. These data are only refined at the county and township level in China, and there are no official statistics on a large sample of villages. Besides, the density of the resident population is frequently used [24,35], but it is only scientifically detected in plain areas. For traditional villages dominated by complex terrains such as mountains and hills, the village area is significantly affected by the terrain, preventing an accurate assessment of the degree of population hollowing. Due to China’s unique dual-track system of household registration and land ownership, the proportion of resident population is an indicator of the number of people who remain in the countryside and reflects the vitality of the rural population [37]. Therefore, this study applied the PRP (percentage of resident population = residents population/household registered population) to measure the degree of population hollowing in traditional villages. This indicator has been successfully tested through many studies [25,37,38]. Using the equal interval method, we further defined the four levels of hollowing associated with the PRP value: no hollowing (≥1.0), mild hollowing (0.7–1.0), moderate hollowing (0.4–0.7), and severe hollowing (<0.4).

### 3.3. The framework of potential impact factor

#### 3.3.1. Factor selection and measurement

The factors influencing rural population hollowing are complex. In terms of nature-related factors, many studies have shown that slope and altitude impact rural population hollowing and changes [39,40]. The winter or summer temperature impacts the rural population’s willingness to migrate [41,42]. Besides, the degree of vegetation coverage is related to population distribution and migration [43,44]. In terms of anthropogenic factors, the number of public service facilities, the number of communication base stations, construction land area, the village’s collective economy, and per capita income significantly impact population hollowing [19,41,45]. In addition, agricultural bases and scenic spots bring industrial economy and employment to villages [19,39]. High-speed rail makes it convenient for villagers to go to cities for work [18]. The inheritance of tangible and intangible culture may increase the resistance of depopulated rural areas [18,46]. Based on these factors related to population hollowing and migration in villages, as well as considering the feasibility of data information collection, this study built the influencing factor framework from nature and anthropogenic aspects, selected 15 indexes, and defined their measurement (Table 1).

**Table 1:** Selection of influencing factors of population hollowing and their measurement.

Criteria	Element	Code	Indicator	Unit	Measurement Method
Natural	Terrain	X1	Slope		Slope of the village
		X2	Altitude	m	Altitude of the village
	Temperature	X3	Summer temperature	°C	Summer surface temperature in the village
		X4	Winter temperature	°C	Winter surface temperature in the village
	Landform	X5	Vegetation index	-	NDVI of the village
Anthropogenic	Daily Life	X6	Numbers of public service facilities	-	The number of facilities within 1 km of the village. The public service facilities specifically include businesses, schools, elderly care, and tourism services.
		X7	Numbers of communication base stations	-	The number of communication base stations within 2 kilometers of the village.
		X8	Area of construction land	acre	Direct extraction
		X9	Distance to the high-speed rail station	km	Distance from village to the high-speed rail station
	Industry	X10	Distance to agricultural bases	km	The shortest distance from the village to agricultural bases, agriculture, including bases of farm, forestry, animal husbandry, and fishery
		X11	Distance to the scenic spots	km	The shortest distance from the village to a scenic spot above the AAA level (defined by Tourist Attraction Rating Categories of China)
	Economy	X12	Village collective income	10,000 CNY	Direct extraction
		X13	Per capita income	10,000 CNY	Direct extraction
	History and culture	X14	The degree of tangible cultural inheritance	-	Quantity of National level*6 + Quantity of Provincial level *3 + Quantity of City level*1 + Quantity of County level*0.5
		X15	The degree of Intangible cultural inheritance	-	Quantity of National level * 6 + Quantity of Provincial level * 3 + Quantity of City level * 1

Note: (1) Data of X7, X12, X13, X14, and X15, were directly collected from the Chinese Traditional Village Survey Registration Form (2017). Except for these, all other data were calculated using GIS 10.6 for spatial calculation.

(2)The calculation of X14 and X15 used the scoring method. According to the classification of Chinese cultural relic protection level and intangible cultural protection level, the number of protection units of corresponding grades were counted respectively, and then were weighted the score of each level and summed (The score of each grade is given from high to low according to the importance of the grade).

#### 3.3.2. Factor stratification method

**Fig 3:**
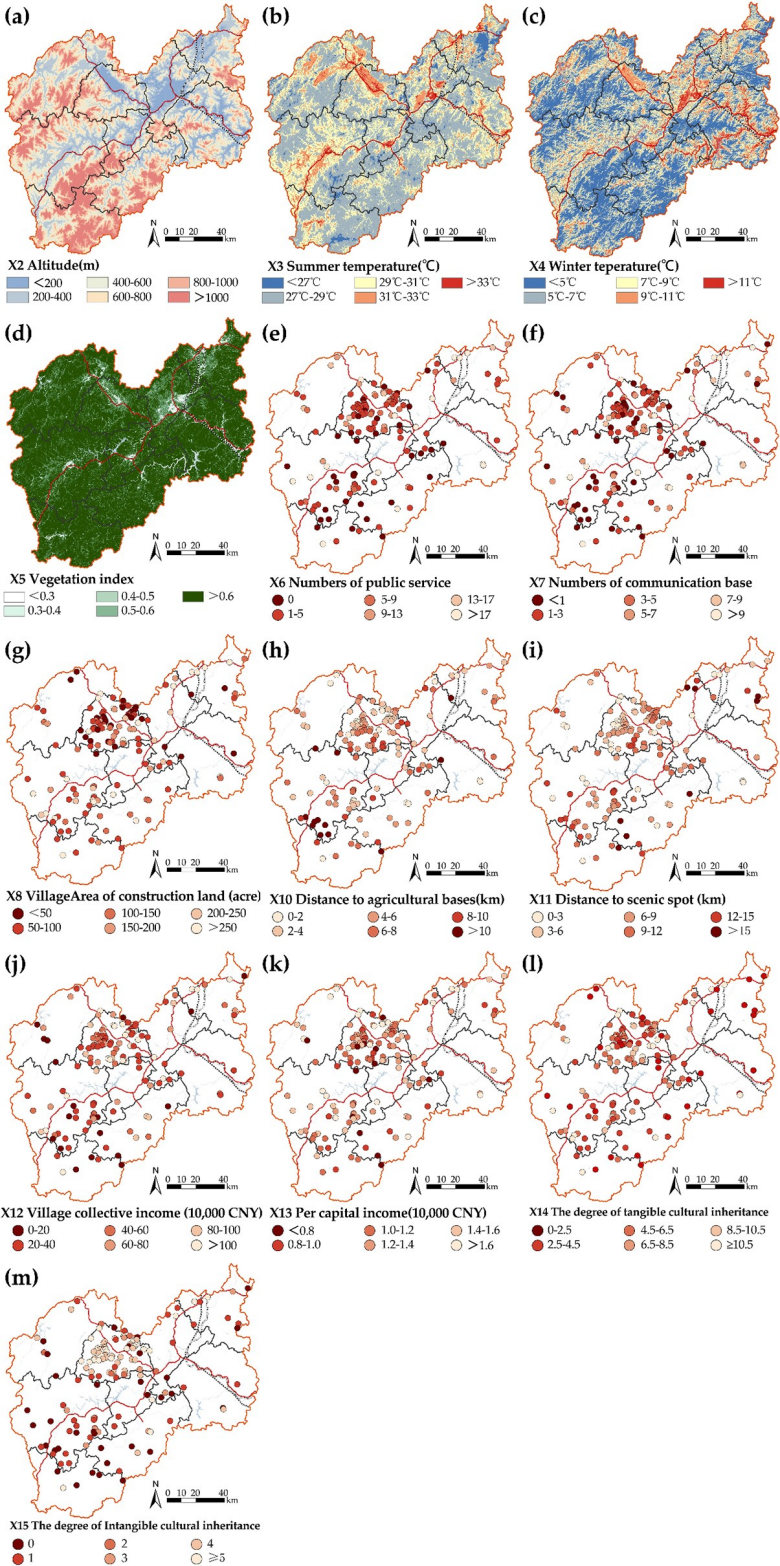
The spatial distributions of all stratified variables. The maps were generated by ArcGIS 10.6 and were for illustrative purposes only.

The independent variables needed to be stratified to be used in the geo-detector. A Spearman correlation analysis was applied to identify 13 correlation factors (X1 and X9 were not correlated). Therefore, this research applied the stratification method to assess the spatial distribution of the population [47], stratified the 13 independent variables using the equal interval method (Table 2), and performed the spatial classification calculation in GIS (Fig 3).

**Table 2:** Stratification of independent variables.

Code	Unit	Stratification Standard					
		1	2	3	4	5	6
X2	m	<200	200–400	400–600	600–800	800–1000	>1000
X3	°C	<27	27–29	29–31	31–33	>33	
X4	°C	<5	5–7	7–9	9–11	>11	
X5	-	<0.3	0.3–0.4	0.4–0.5	0.5–0.6	>0.6	
X6	-	0	1–5	5–9	9–13	13–17	>17
X7	-	0	1–3	3–5	5–7	7–9	>9
X8	acre	<50	50–100	100–150	150–200	200–250	>250
X10	km	0–2	2–4	4–6	6–8	8–10	>10
X11	km	0–3	3–6	6–9	9–12	12–15	>15
X12	10,000 CNY	0–20	20–40	40–60	60–80	80–100	>100
X13	10,000CNY	<0.8	0.8–1	1–1.2	1.2–1.4	1.4–1.6	>1.6
X14	-	0–2.5	2.5–4.5	4.5–6.5	6.5–8.5	8.5–10.5	≥10.5
X15	-	0	1	2	3	4	≥5

Note: The names of the indicators corresponding to each code are shown in Table 1.

### 3.4. Global autocorrelation analysis

The Global Moran’s I was used to determine if the disparities in PRP were space-related [48]. This index reflects the similarity of the attribute values of the unit adjacent to or near space, then judges the spatial distribution pattern of the whole area. The formula is:

$$I=\frac{n}{\sum_{i=1}^{n}\sum_{j=1}^{n}W_{ij}}\frac{\sum_{i=1}^{n}\sum_{j=1}^{n}W_{ij}(x_{i}-\bar{x})(x_{j}-\bar{x})}{\sum_{i=1}^{n}{\left(x_{i}-\bar{x}\right)}^{2}}$$ (1)

where I represents the global Moran’s I index; n represents the number of village units; xi and xj are the PRP value of the i-th and j-th administrative units, respectively; x is the average PRP value for all administrative units; and w represents the binary weight that was determined using the Queen-based adjacency method. The Global Moran’s I index ranges from -1 to 1. Values greater than 0 indicated a positive spatial correlation, whereas values less than 0 indicated a negative spatial correlation. The larger the absolute value was, the higher the degree of spatial correlation was.

### 3.5. Geo-detector

The key assumption with the geo-detector is that if geographic factor a is controlled by another geographic factor b, then b will show a spatial distribution similar to a [28]. The model does not need to follow the restrictive assumptions of traditional statistical methods, nor does it involve complex parameter setting procedures [49]. In this method, the factor detection can identify the influencing factors; the interactive detection can explain the interaction of pair factors on the dependent variable, and the risk area detection can identify the factor’s risk interval. This makes it an effective tool to study the mechanisms driving complex geographic factors [50].

(1) Influencing factor detection. The value of q was used to measure the explanatory power of the factors. The specific formula is as follows [28]:

$$q=1-\frac{\sum_{h=1}^{L}N_{h}\sigma_{h}^{2}}{{N\sigma }^{2}}$$ (2)

In the formula: h = 1, …, L is the stratum of factor X; N_h_ represents the number of samples in stratum h; N denotes the total number of spatial units (in this case, traditional villages); and σ h and σ are the variance of samples in stratum h and the total variance, respectively. The value of q means that X explains 100×q% of Y, with a value range of [0, 1]. The larger the value of q was, the greater its influence on Y was.

(2) Interaction detection. This method analyzes the interaction between different influencing factors, evaluating whether the interaction increased or decreased the influence on the spatial disparity of the dependent variable. To detect the interaction of factors, e.g., X1 and X2, first, the values of q for the two factors were calculated using Eq (2). Then, the layers of X1 and X2 were superimposed to form the new layer X1∩X2 (∩denotes the interaction between factor X1and X2), generating the values of q_(X1∩X2)_ based on their interaction. There were five categories of relationships generated by comparing the interaction q value of the pair factors and the q value of each one, as shown in **Table 3**.

**Table 3:** Interaction categories of two factors and the interaction relationship.

Description	Interaction
q(X1∩X2)<Min(q(X1),q(X2))	Weaken; nonlinear
Min(q(X1),q(X2))<q(X1∩X2)<Max(q(X1),q(X2))	Weaken; unique
q(X1∩X2)>Max(q(X1),q(X2))	Enhanced, bivariate
q(X1∩X2) = q(X1)+q(X2)	Independent
q(X1∩X2)>q(X1)+q(X2)	Enhanced, nonlinear

(3) Risk area detection. The result of the risk detector reflected how each independent variable affected the dependent variable in different attribute intervals. A t-test was used to compare the average difference between different stratum. The larger the difference was, the greater the threat of population hollowing in the area was. The t-statistic formula is as follows:

$$t_{{\bar{y}}_{h-1}{\bar{y}}_{h-2}}=\frac{{\bar{Y}}_{h=1}-{\bar{Y}}_{h=2}}{{\left[\frac{V_{ar}({\bar{Y}}_{h-1})}{n_{h-1}}+\frac{V_{ar}({\bar{Y}}_{h-2})}{n_{h-2}}\right]}^{1/2}}$$ (3)

In the formula, ${\bar{Y}}_{h}$ is the average value of Y in the h stratum; nh is the number of units in stratum h; and Var represents the variance.

## 4. Results

### 4.1. Features of traditional villages’ SDPH

**Fig 4:**
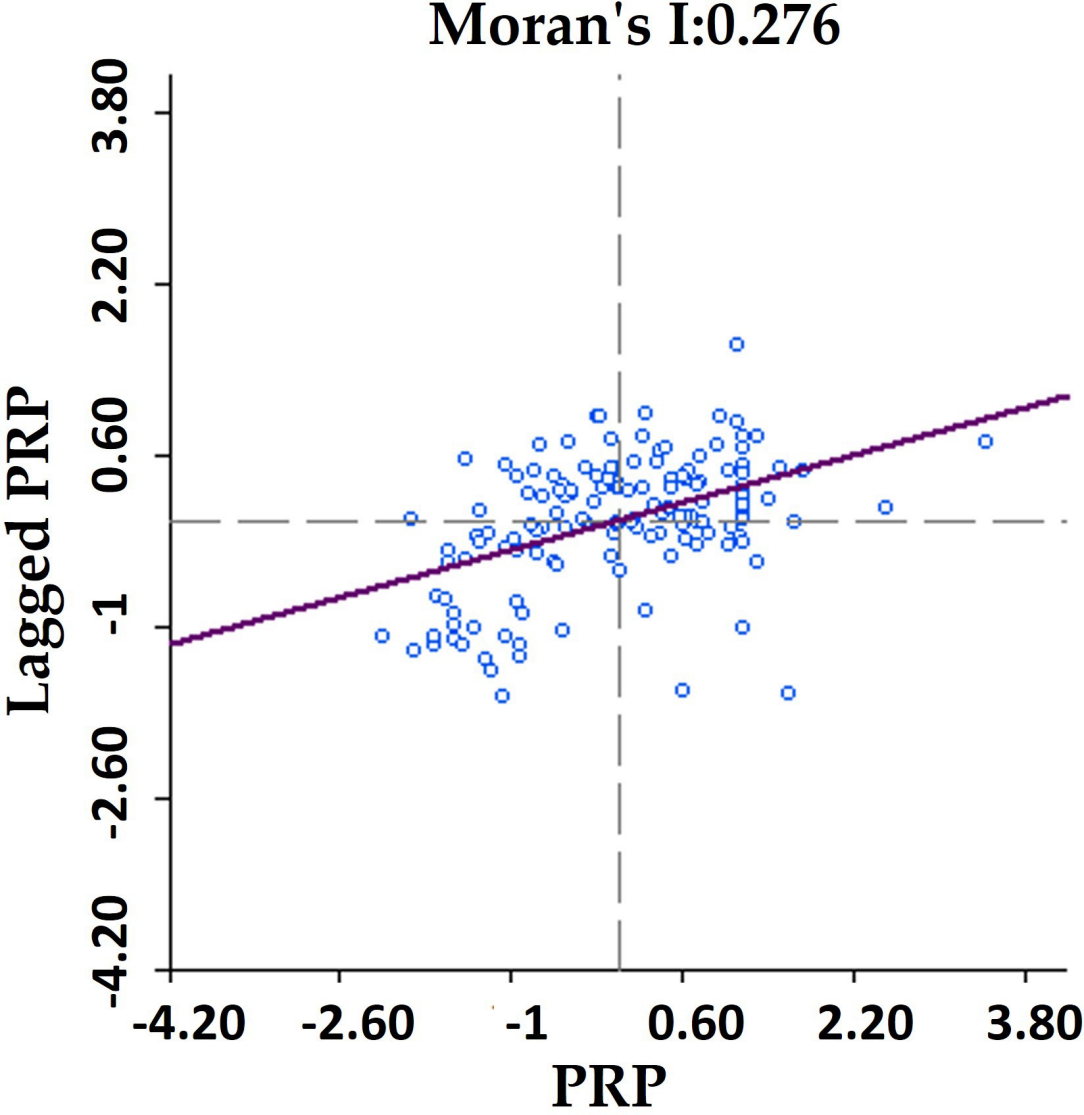
Moran scatter plot of PRP values.

**Fig 5:**
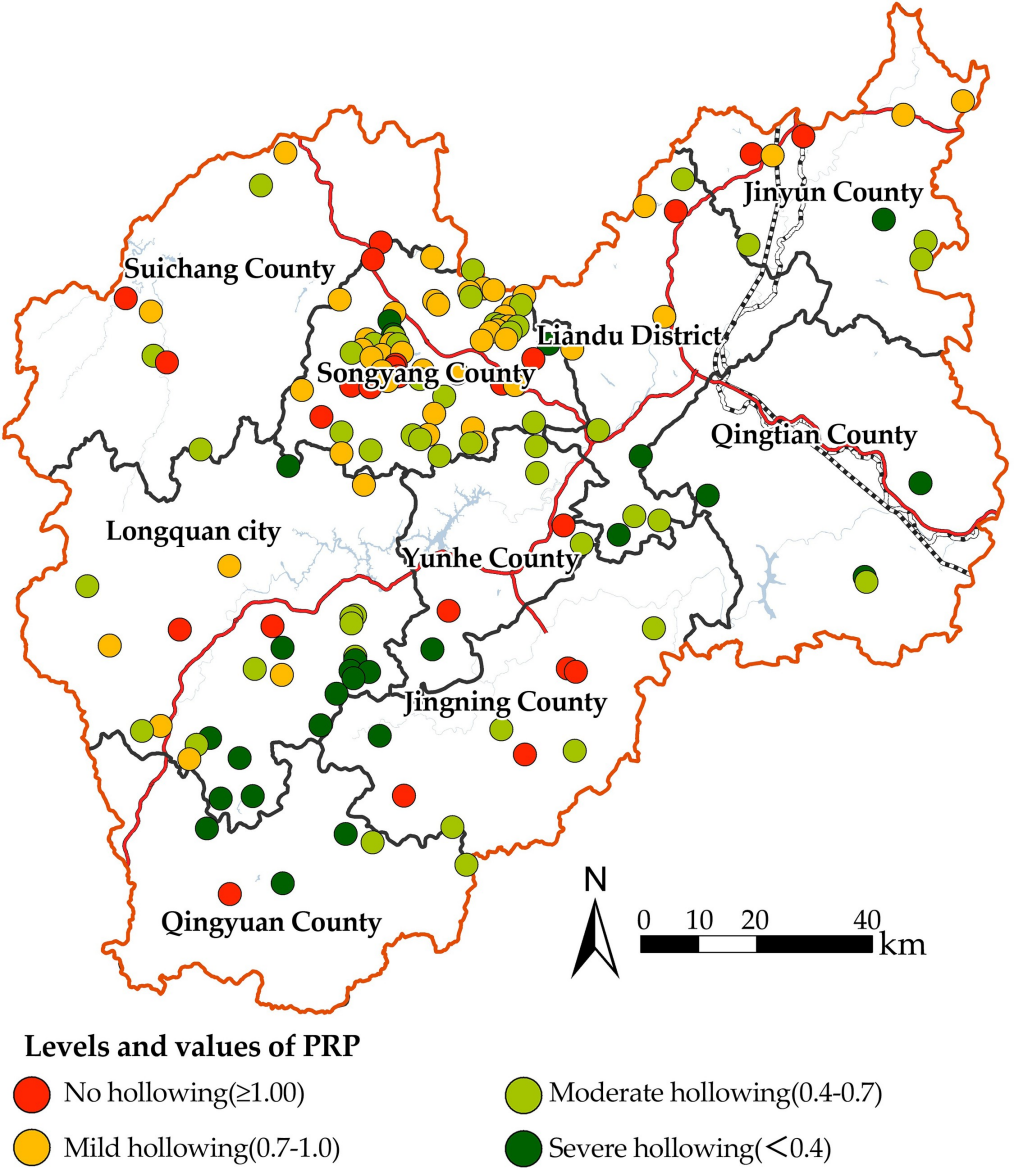
Spatial distribution of PRP values for traditional villages in Lishui area. The maps were generated by ArcGIS 10.6 and were for illustrative purposes only.

Table 4 showed that the PRP in traditional villages in Lishui exhibited significant polarization, with the interval value from 0.05 to 4.14. There are 82.67% showed more than mild hollowing features, with a relatively high proportion of villages showed moderate hollowing (33.33%). The global autocorrelation analysis of PRP (Fig 4) led to the finding that Moran I = 0.276. The significance test identified that Z = 5.7645, with a P<0.01, indicating a spatially significant positive correlation. We further analyzed the PRP values spatially and visually (Fig 5), and found there were significant spatial disparities in the PRP values of traditional villages in Lishui: (1) PRP values were higher in the north and lower in the south, and there were significant differences in the mean value of the PRP between the north (0.73) and south (0.57). (2) Significant hollowing areas were distributed at the junction of Longquan, Qingyuan, and Jingning counties in the south. Traditional villages in the central Songyang county and northern Jinyun county in the north had a high proportion of PRP, with relatively light hollowing.

**Table 4:** PRP values and population hollowing levels in traditional villages.

Level of Population Hollowing	Interval Value of PRP	Mean Value of PRP	Number	Percentage(%)
No hollowing	≥1.0	1.07	26	17.33
Mild hollowing	0.7–1.0	0.84	48	32.00
Moderate hollowing	0.4–0.7	0.55	50	33.33
Severe hollowing	<0.4	0.26	26	17.33
Total	0.05–4.14	0.68	150	100
North	0.12–1.64	0.73	98	66.22
South	0.05–1.37	0.57	50	33.78

**Note:** The southern area includes four counties: Longquan, Qingyuan, Jingning, and Yunhe. The northern area includes five counties: Suichang, Songyang, Liandu, Qingtian, and Jinyun.

### 4.2. Correlation factor identification

A Spearman’s correlation analysis (Table 5) found that the PRP in traditional villages was not related to the slope and the distance to the high-speed rail station. Screening did identify 13 other significantly more factors, which all passed the test of variance inflation factor.

**Table 5:** Correlation analysis between PRP and each potential factor.

Code	X1	X2	X3	X4	X5	X6	X7	X8	X9	X10	X11	X12	X13	X14	X15
**Correlation Coefficient**	-0.112	-0.425**	0.256**	0.296**	-0.284**	0.382**	0.317**	0.216**	-0.082	-0.235**	-0.328**	0.294**	0.183*	0.307**	0.331**
**Significance**	0.173	0.000	0.002	0.000	0.000	0.000	0.000	0.008	0.321	0.004	0.000	0.000	0.025	0.000	0.000
VIF	1.437	1.932	2.184	1.775	1.459	4.888	3.975	2.351	1.614	1.549	1.427	2.462	1.240	1.944	1.206

* Significant at the 5% level (two-tail).

**Significant at the 1% level (two-tail).

### 4.3. Geo-detector analysis

#### 4.3.1. Factor detector analysis

Among the 13 correlated factors, 12 factors with different degrees of influence were identified (Table 6). The factor, winter temperature, fell short of statistical significance impact on PRP value (p > 0.1). However, elevation, the number of public service facilities, the number of communication base stations, and the vegetation index were significant at a 0.01 level, with the largest explanatory power for PRP (17.7%, 17.6%, 16.9%, and 15.3%, respectively). Summer temperature; area of construction land; distance to agricultural bases; per capita income; and the degree of tangible cultural inheritance were significant at a 0.05 level. The distance from the scenic spot and the degree of intangible cultural inheritance have certain trends toward significance, with the smallest explanatory power, at 7.9% and 8.7%, respectively. Therefore, both natural and anthropogenic factors have important influences on the spatial disparity in the PRP in Lishui. The influence of the degree of tangible cultural inheritance was not the highest (10.0%), but was close to those economic and industrial factors and exceeded the degree of intangible cultural inheritance (8.7%) in the similar category.

**Table 6:** Explanatory power of influencing factors to dependent variables.

Code	q	p	rank	Code	q	p	rank
X2	0.177***	0.000	1	X6	0.176***	0.005	2
X3	0.085**	0.049	11	X7	0.169***	0.000	3
X4	0.042	0.441	-	X8	0.119**	0.022	5
X5	0.153**	0.011	4	X10	0.092**	0.023	7
				X11	0.079*	0.080	12
				X12	0.096*	0.068	9
				X13	0.095**	0.034	8
					X14	0.100**	0.033	6
				X15	0.087*	0.084	10

* p-value< 0.1

** p-value < 0.05

***p-value < 0.01.

#### 4.3.2. Interaction detector analysis

Among the 12 influencing factors, 66 pairs of interaction effects were obtained using the interaction detector, as shown in Table 7. In 83.33% of cases, the explanatory power of the interaction between pair factors was higher than the sum of the explanatory power of two single factors. This indicated a nonlinear enhancement effect. Although the degree of intangible cultural inheritance (X15) had a small influence on its own (8.4%), it had the largest explanatory power (q = 0.679) in the interaction with the altitude (X2). It also enhanced the explanatory power to a relatively high level when it interacted with the number of public service facilities, the distance from the scenic spot, and the number of communication base stations, with q values of 0.586, 0.546, and 0.415, respectively.

**Table 7:** Interaction between pair factors.

MAX(A,B)	C	A+B	results	Interpretation	MAX(A,B)	C	A+B	results	Interpretation
X2 = 0.177<	X2∩X3 = 0.344	0.262 = X2+X3	C>A+B	↑↑	X6 = 0.176<	X6∩X11 = 0.226	0.345 = X6+X11	C<A+B	↑
X2 = 0.177<	X2∩X5 = 0.379	0.330 = X2+X5	C>A+B	↑↑	X6 = 0.176<	X6∩X12 = 0.276	0.272 = X6+X12	C>A+B	↑↑
X2 = 0.177<	X2∩X6 = 0.320	0.352 = X2+X6	C<A+B	↑	X6 = 0.176<	X6∩X13 = 0.277	0.271 = X6+X13	C>A+B	↑↑
X2 = 0.177<	X2∩X7 = 0.299	0.296 = X2+X7	C>A+B	↑↑	X6 = 0.176<	X6∩X14 = 0.361	0.276 = X6+X14	C>A+B	↑↑
X2 = 0.177<	X2∩X8 = 0.326	0.268 = X2+X8	C>A+B	↑↑	X6 = 0.176<	X6∩X15 = 0.586	0.263 = X6+X15	C>A+B	↑↑
X2 = 0.177<	X2∩X9 = 0.258	0.255 = X2+X9	C>A+B	↑↑	X7 = 0.119<	X7∩X8 = 0.276	0.211 = X7+X8	C>A+B	↑↑
X2 = 0.177<	X2∩X11 = 0.274	0.346 = X2+X11	C<A+B	↑	X7 = 0.120<	X7∩X9 = 0.276	0.198 = X7+X9	C>A+B	↑↑
X2 = 0.177<	X2∩X12 = 0.251	0.273 = X2+X12	C<A+B	↑	X7 = 0.121<	X7∩X11 = 0.268	0.289 = X7+X11	C<A+B	↑
X2 = 0.177<	X2∩X13 = 0.326	0.272 = X2+X13	C>A+B	↑↑	X11 = 0.169<	X7∩X12 = 0.27	0.216 = X7+X12	C>A+B	↑↑
X2 = 0.177<	X2∩X14 = 0.412	0.277 = X2+X14	C>A+B	↑↑	X7 = 0.121<	X7∩X13 = 0.336	0.214 = X7+X13	C>A+B	↑↑
X2 = 0.177<	X2∩X15 = 0.679	0.264 = X2+X15	C>A+B	↑↑	X7 = 0.122<	X7∩X14 = 0.333	0.22 = X7+X14	C>A+B	↑↑
X5 = 0.153<	X3∩X5 = 0.200	0.239 = X3+X5	C<A+B	↑	X7 = 0.123<	X7∩X15 = 0.345	0.207 = X7+X15	C>A+B	↑↑
X6 = 0.176<	X3∩X6 = 0.388	0.261 = X3+X6	C>A+B	↑↑	X8 = 0.092<	X8∩X9 = 0.209	0.170 = X8+X9	C>A+B	↑↑
X7 = 0.119<	X3∩X7 = 0.265	0.205 = X3+X7	C>A+B	↑↑	X11 = 0.169<	X8∩X11 = 0.285	0.261 = X8+X11	C>A+B	↑↑
X8 = 0.092<	X3∩X8 = 0.265	0.177 = X3+X8	C>A+B	↑↑	X12 = 0.096<	X8∩X12 = 0.231	0.188 = X8+X12	C>A+B	↑↑
X3 = 0.085<	X3∩X9 = 0.336	0.164 = X3+X9	C>A+B	↑↑	X13 = 0.095<	X8∩X13 = 0.214	0.187 = X8+X13	C>A+B	↑↑
X11 = 0.169<	X3∩X11 = 0.369	0.254 = X3+X11	C>A+B	↑↑	X14 = 0.100<	X8∩X14 = 0.239	0.192 = X8+X14	C>A+B	↑↑
X12 = 0.096<	X3∩X12 = 0.231	0.182 = X3+X12	C>A+B	↑↑	X8 = 0.092<	X8∩X15 = 0.247	0.179 = X8+X15	C>A+B	↑↑
X13 = 0.095<	X3∩X13 = 0.206	0.18 = X3+X13	C>A+B	↑↑	X11 = 0.169<	X9∩X11 = 0.232	0.248 = X9+X11	C<A+B	↑
X14 = 0.100<	X3∩X14 = 0.217	0.186 = X3+X14	C>A+B	↑↑	X12 = 0.096<	X9∩X12 = 0.206	0.175 = X9+X12	C>A+B	↑↑
X15 = 0.087<	X3∩X15 = 0.323	0.173 = X3+X15	C>A+B	↑↑	X13 = 0.095<	X9∩X13 = 0.202	0.174 = X9+X13	C>A+B	↑↑
X6 = 0.176<	X5∩X6 = 0.434	0.329 = X5+X6	C>A+B	↑↑	X14 = 0.100<	X9∩X14 = 0.263	0.179 = X9+X14	C>A+B	↑↑
X7 = 0.121<	X5∩X7 = 0.258	0.273 = X5+X7	C>A+B	↑↑	X15 = 0.087<	X9∩X15 = 0.546	0.166 = X9+X15	C>A+B	↑↑
X8 = 0.092<	X5∩X8 = 0.275	0.245 = X5+X8	C>A+B	↑↑	X11 = 0.169<	X11∩X12 = 0.246	0.265 = X11+X12	C<A+B	↑
X5 = 0.153<	X5∩X9 = 0.464	0.232 = X5+X9	C>A+B	↑↑	X11 = 0.170<	X11∩X13 = 0.274	0.264 = X11+X13	C>A+B	↑↑
X11 = 0.169<	X5∩X11 = 0.353	0.322 = X5+X11	C>A+B	↑↑	X11 = 0.171<	X11∩X14 = 0.371	0.269 = X11+X14	C>A+B	↑↑
X12 = 0.096<	X5∩X12 = 0.463	0.25 = X5+X12	C>A+B	↑↑	X11 = 0.172<	X11∩X15 = 0.415	0.256 = X11+X15	C>A+B	↑↑
X13 = 0.095<	X5∩X13 = 0.239	0.248 = X5+X13	C<A+B	↑	X12 = 0.096<	X12∩X13 = 0.224	0.191 = X12+X13	C>A+B	↑↑
X14 = 0.100<	X5∩X14 = 0.242	0.254 = X5+X14	C<A+B	↑	X14 = 0.100<	X12∩X14 = 0.225	0.197 = X12+X14	C>A+B	↑↑
X15 = 0.087<	X5∩X15 = 0.331	0.241 = X5+X15	C>A+B	↑↑	X12 = 0.096<	X12∩X15 = 0.261	0.184 = X12+X15	C>A+B	↑↑
X6 = 0.176<	X6∩X7 = 0.277	0.295 = X6+X7	C<A+B	↑	X14 = 0.100<	X13∩X14 = 0.276	0.195 = X13+X14	C>A+B	↑↑
X6 = 0.176<	X6∩X8 = 0.298	0.267 = X6+X8	C>A+B	↑↑	X13 = 0.095<	X13∩X15 = 0.355	0.182 = X13+X15	C>A+B	↑↑
X6 = 0.176<	X6∩X9 = 0.336	0.254 = X6+X9	C>A+B	↑↑	X14 = 0.100<	X14∩X15 = 0.225	0.188 = X14+X15	C>A+B	↑↑

**Note:** The symbol ‘∩’ denotes the intersection between A and B. “↑” means that x1 and x2 are mutually enhanced, and “↑↑” means that x1 and x2 are non-linearly enhanced. The numbers in the table are q statistics.

#### 4.3.3. Risk detector analysis

**Fig 6:**
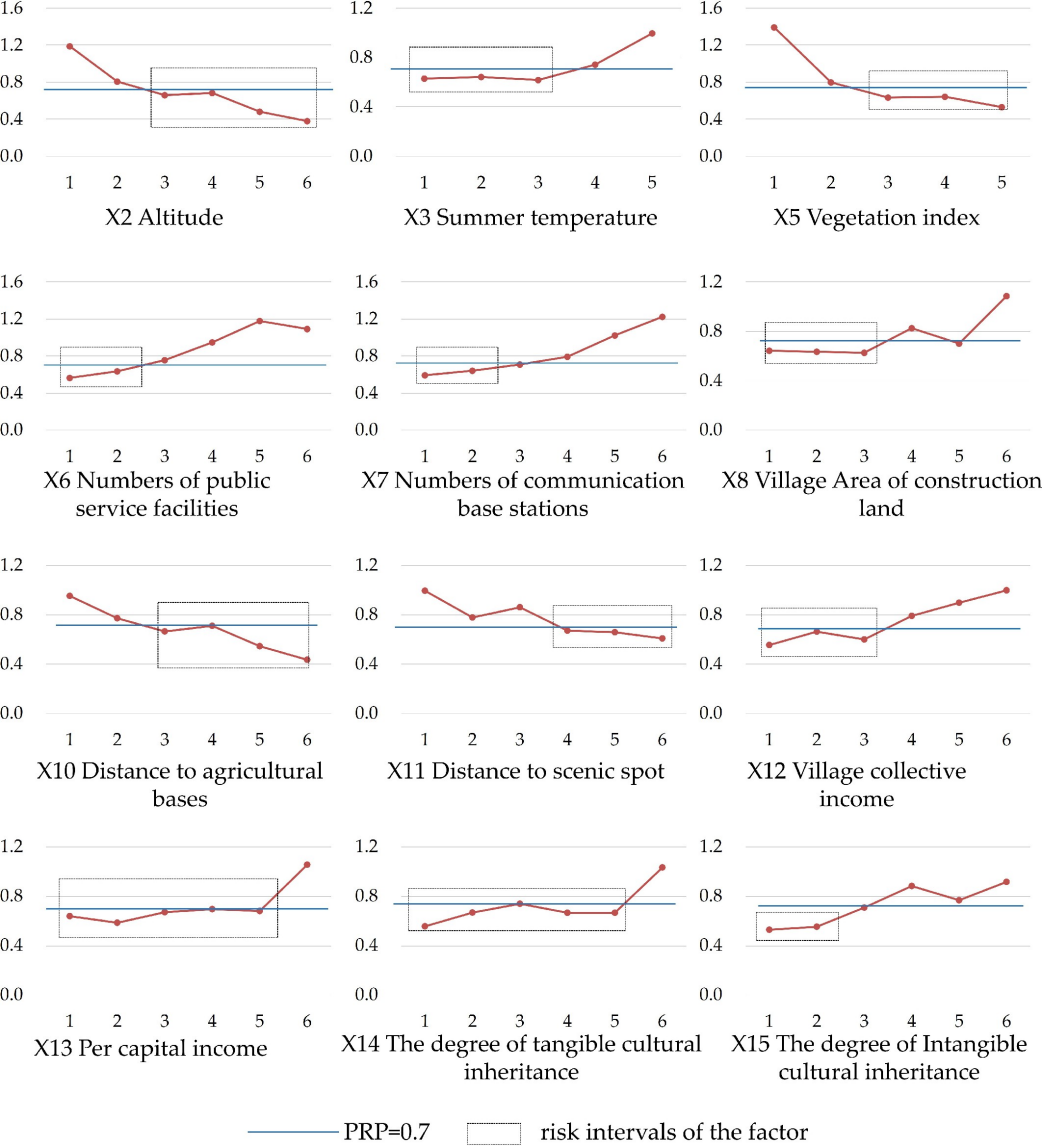
Changes of the average PRP value of each factor at different strata.

The risk detector analysis was used to generate the average PRP of each stratum for the factors, and the significance of their differences in terms of influence. The classification of the independent variable data in Table 2 shows that each stratum of each factor had a corresponding average PRP value. Using elevation as an example, the PRP average values of the six strata of 1, 2, 3, 4, 5, and 6 were 1.19, 0.81, 0.66, 0.68, 0.48, and 0.38, respectively. The results of other factors were also be obtained. A PRP change chart (Fig 6) was drawn based on strata data, which was used to determine the influencing trend and the risk area. This study defined the risk area of each influencing factor based on the strata interval obtained when the average PRP was less than 0.7. This was based on the degree of hollowing (that is, when PRP< 0.7, the population hollowing is moderate or severe), which was defined in section 3.2. This implied that certain factors generate a high risk of population hollowing in villages within a certain attribute range. This method was originally used to detect disease risk areas [51], and has also been successfully used to detect risk areas for air pollution [52].

Fig 6 shows that most factors had almost linear effects on PRP. The PRP was negatively affected by a small number of factors (including elevation, vegetation index, distance from the agricultural base, and distance from the scenic spot), but was positively affected by most of the other factors. Combined with Fig 6 and Table 2, this research generated factor-specific risk intervals. If the attribute of each factor of the village fell in these intervals, it indicated a risk of population hollowing above a moderate level. These intervals were as follows: lower than 31°C for the average summer temperature; less than 5 facilities within 1 km; less than 3 communication base stations within 2 km; less than 600,000 yuan for the village’s collective income; less than 10,000 yuan for the per capita income; higher than 600 m for the altitude; greater than 0.3 for the NDVI; more than4 km for the distance to agricultural bases; and more than 9 km for the distance to a scenic spot. Based on further integration with the measurement of cultural factors (Table 1), we also determined two cultural factors’ risk intervals as follows: if the evaluation point of the degree of material cultural heritage was less than 4.5, that is, there are no national cultural relics, and there are less than two provincial-level cultural relics; or if the evaluation score concerning the degree of intangible cultural heritage is less than or equal to 1, that is, the number of intangible cultural relics at or above the municipal level is less than 2, there is a risk of the population hollowing above a moderate level.

## 5. Discussion

### 5. 1 Key natural and anthropogenic driving factors

This study used PRP values to characterize population hollowing, and found that natural and anthropogenic factors influence SDPH in traditional villages in Lishui. In terms of natural factors, altitude was the most significant factor and can affect the types of crops, yields, and production and living conditions of farmers [17,39,40]. Similar to ordinary villages, higher altitudes of the traditional villages are associated with more difficult production and living conditions. As a result, more people leave for employment, leading to a lower PRP. The vegetation index also significantly negatively impacted the PRP. Areas with high vegetation coverage are strictly protected to alleviate the destruction of nature by urban construction in eastern China [53]. This somewhat limits the economic development of these villages. As a result, a higher local vegetation index is associated with more restricted economic development and a lower PRP. Also, in the Philippines, the high surface temperature in summer affects agricultural cultivation, causing an increased population migration rate [42]. However, in the case area, the average surface temperature of the traditional villages in summer was 30.94°C, which does not negatively impact local agricultural production. The reason that the summer temperature had a slightly positive impact on the PRP may be related to the general trend that the population in the Yangtze River Delta, where Lishui is located, is gathering in areas with high summer temperatures, caused by high urbanization [54].

In terms of anthropogenic factors, (1) the factors associated with daily life had significant explanatory power. The number of public service facilities and the number of communication base stations had critical positive explanatory power. This may be because for the traditional villages, which generally lack the facilities needed for basic life, lower facility density or few communication base stations will notably lower life satisfaction and convenience, driving residents towards living in towns or cities [11,55]. In addition, the positive explanatory power of the area of construction land was great. This may be because the living area of existing traditional buildings in the village is generally limited, as the protection policy makes it difficult to rebuild or expand these houses. Thus, it cannot meet villages’ basic demands for expanded living areas, driving them to move to places with more abundant living space.

(2) At the industrial level, agricultural bases and scenic spots can bring employment opportunities to villagers and increase villagers’ income. As traditional villages are generally dominated by agro-farming, and the development of rural tourism relying on scenic spots lags, the influence of agricultural bases on employment and income is generally greater compared to the influence of scenic spots. This may result in there being a relatively higher explanatory power for the factor of distance to agricultural bases.

(3) At the economic level, a village’s lower collective income may result in fewer job opportunities for villages [19,25]. Lower per capita income is also associated with larger differences in income between urban and rural residents. These two factors increase the drive for villagers to go to urban areas for more jobs and higher income.

(4) At the historical and cultural level, relevant factors also have some explanatory power for the PRP, highlighting an important innovative contribution of this study. The degree of material and cultural inheritance had positive exploratory power. It may be because a village with a better tangible cultural inheritance is associated with a higher level and many preserved cultural relics. It also generally has more funds to protect cultural relics and more attractive historical tourist sites. In this way, villagers may be more likely to experience improved living conditions and income from tourism services, and may be more willing to stay in the village to live and work. Further, the degree of intangible cultural inheritance may affect the PRP from the perspective of village identity. A low level of intangible cultural inheritance may weaken the social network, community associations, and the residents’ sense of belonging to the village [11,46]. This is consistent with the American rural studies, showing that community cultural attachment has an important impact on residents’ willingness to leave [23].

### 5.2 Strategic recommendation for preventing population loss

**Fig 7:**
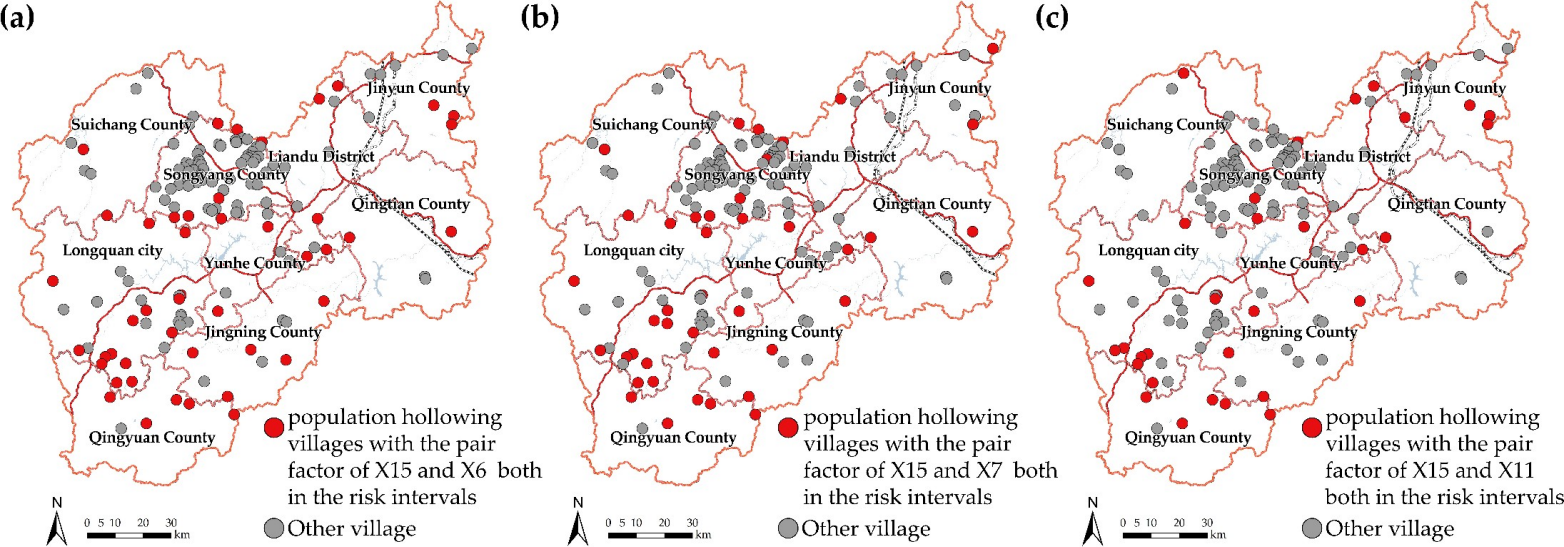
Population hollowed villages with high-influence interaction pair factors both in the risk intervals. (The degree of intangible cultural inheritance (X15) interacts with the number of public service facilities(X6), number of communication base stations(X7), distance to scenic spot(X11), respectively). The maps were generated by ArcGIS 10.6 and were for illustrative purposes only.

In implementing strategies to prevent population hollowing, the explanatory power of the influencing factors on the spatial disparity of PRP should be considered first. For natural factors, elevation and summer temperature are difficult to change, and reducing vegetation coverage is unsustainable for village development. Therefore, it is recommended that villages focus on anthropogenic factors, especially the three anthropogenic-level interaction factor pairs that have the highest explanatory power. Coupled with the risk detector results, the population hollowed villages with these three pairs of factors meeting the criteria of the risk intervals can be identified separately (**Fig 7**), where each pair of factors could be improved together to effectively prevent population loss. That is, PRP is expected to increase if the intangible cultural inheritance increased, while also coordinating an increase in the construction of public service facilities, or expanding the communication base stations, or developing scenic spots in adjacent areas.

### 5.3 Contributions and future work

Retaining labor for traditional villages is the foundation of protection and inheritance, highlighting the significance of this study towards reviving traditional culture. There are two main contributions of this work: (1) In exploring the driving factors, both historical and cultural factors were integrated to construct a framework of potential influencing factors of SDPH suitable for traditional village characteristics. (2) An empirically tested approach from agglomeration analysis, correlation analysis to the geo-detector method was constructed, which can be applied to effectively identify the impact factors that influence SDPH of traditional villages in a prefecture-level city and to explore the interaction between factors and the factors’ risk intervals.

In China, even among cities located in the same province, there are great differences in supporting policies and funds for the revitalization of traditional villages. These differences will have direct impacts on the population hollowing of traditional villages [56]. Thus, if this method needs to be applied to a larger area (such as a region with multiple cities), the policy and subsidy differences between cities need to be incorporated into the impact factor framework and to be quantified reasonably. We hope to take this as a breakthrough direction in the future, continuing to combine municipal policies and subsidy factors into the method, to explore the key factors that influence the SDPH of traditional villages within multiple cities.

## 6. Conclusion

Incorporating historical and cultural factors, this study explored an approach to quantitatively evaluate the factors influencing the SDPH of traditional villages at a prefecture-level city scale. 150 national traditional villages in the Lishui area were conducted for the experiment and their complicated nature of the SDPH was revealed as followed. (1) Altitude, the number of public service facilities, and the number of communication base stations were the three most significant factors. (2) The influence produced the highest when the degree of intangible cultural inheritance interacted with altitude. (3) The factor intervals leading to the population hollowing above a moderate degree could be detected using the risk detector.

The research findings of this study show that the method effectively analyzed the influencing factors, after distinguishing the SDPH of traditional villages. It also had the feasibility to reveal the interaction of two factors on the population hollowing and the factors’ risk intervals. These experimental results provide a basis for preventing population loss in the case study area and also highlight a new analytical method that could be applied to other single prefectural cities with traditional villages’ SDPH issues.
